# The antibiotic resistance proteome of *Acinetobacter baumanii* MDR isolate MMC#4

**DOI:** 10.1186/1471-2105-13-S12-A20

**Published:** 2012-07-31

**Authors:** Dana Marshall, Jianan Dong, Leon Dent, Siddharth Pratap

**Affiliations:** 1 Department of Pathology, Anatomy and Cell Biology, Meharry Medical College, Nashville, TN 37208, USA; 2 Bioinformatics and Proteomics Core, Meharry Medical College, Nashville, TN 37208, USA; 3 Department of Surgery, Meharry Medical College, Nashville, TN 37208, USA

## Background

Two hundred and forty-seven isolates of *Acinetobacter baumanii* (AB) were identified in the Nashville General Hospital at Meharry epidemiology database for a three year period. Of these isolates, 77% were multi-drug resistant (MRAB). Mechanical ventilation and multiple site recovery were associated with MRAB, and MRAB isolates were associated with increased mortality relative to sensitive AB isolates [1]. AB acquires resistance rapidly and the mechanisms are still being identified. Proteomic analysis can identify proteins that change in their expression levels in the presence of antibiotics, a possible mechanism of resistance for AB. No proteome databases exist for this organism so antibiotic sensitivity and resistance proteomes were generated for MRAB bronchial wash isolate MMC#4.

## Materials and methods

AB MMC#4 was grown in plain LB broth or LB broth supplemented with MIC50 concentrations of levofloxacin, tobramycin, gentamicin, cefotaxime and meropenem. Cell pellets were lysed and total protein run on an SDS-PAGE gel. Protein bands were excised and in-gel digested with trypsin. Resulting peptides were analyzed using a Thermo Finnigan LTQ ion trap mass spectrometer equipped with a 1-D nanoLC pump (Eksigent), Nanospray source (James A Hill Company), and Xcalibur 2.0 SR2 instrument control (Thermo Scientific). Peptides were separated on a packed capillary tip (Polymicro Technologies, 50 μm ID X 9 cm) with C18 reverse phase resin (5 μm, 300 Å, Phenomenex). Tandem spectra were acquired using a data dependent scanning mode in which one full MS scan (m/z 200-2000) was followed by 5 MS-MS scans. Tandem spectra were searched against the NCBI AB strain ACICU database using MryiMatch and IDPicker software. The search database was concatenated with the reverse sequences to determine false discovery rates. Proteins identified by less than two peptide spectral were eliminated (FDR <5%) and the output was filtered using IDPicker using a false positive ID threshold of 5%. Protein reassembly from identified peptide sequences was done as described by Zhang et al. [2].

## Results and conclusions

This analysis resulted in the identification of 125 high-confidence hits. Ten of these are presented in Table 1. Antibiotic stress resulted in increased detection of beta-lactamase (cefotaxime is a beta-lactam antibiotic) as well as several proteins associated with oxidative stress that have not previously been described in the context of MRAB resistance mechanisms. These results reinforce the utility of proteomes of antibiotic resistance for MRAB isolates in the identification of potential diagnostic and therapeutic targets, as well as resistance mechanisms, for this emerging pathogen.

**Table 1:** Top 10 peptides identified more frequently in AB grown in the presence of antibiotic pressure when compared to AB grown in the absence of antibiotic pressure.

Proteins	Number of Hits Antibiotic Treated	Number of Hits No Antibiotics
superoxide dismutase [Acinetobacter baumannii ACICU]	40	1
outer membrane protein, related peptidoglycan-associated (lipo)protein [Acinetobacter baumannii ACICU]	9	2
gluconate kinase [Acinetobacter baumannii ACICU]	8	2
electron transfer flavoprotein subunit alpha [Acinetobacter baumannii ACICU]	7	3
Zn-dependent alcohol dehydrogenase, class III [Acinetobacter baumannii ACICU]	2	1
putative porin protein associated with imipenem resistance [Acinetobacter baumannii ACICU]	3	2
hypothetical protein ACICU_02436 [Acinetobacter baumannii ACICU]	76	53
beta-lactamase [Acinetobacter baumannii ACICU]	452	325
malate dehydrogenase [Acinetobacter baumannii ACICU]	50	38
chaperonin GroEL [Acinetobacter baumannii ACICU]	50	43
